# Short-duration ischemic preconditioning does not improve jump or change of direction performance in elite male handball players

**DOI:** 10.1371/journal.pone.0334747

**Published:** 2025-10-16

**Authors:** Okan Kamiş, Neslihan Akçay, Kadir Keskin, Nicholas Rolnick, Carlos García Sánchez, Victor S. de Queiros, Fatih Harun Turhan, João Guilherme Vieira, Alexander Montoye, Rodrigo Ramalho Aniceto

**Affiliations:** 1 Department of Sports and Health, Aksaray University, Aksaray, Türkiye; 2 School of Sport, Exercise and Rehabilitation, Northumbria University, Newcastle upon Tyne, United Kingdom; 3 Faculty of Sport Sciences, Karabük University, Karabük, Türkiye; 4 Faculty of Sports Sciences, Gazi University, Ankara, Türkiye; 5 Department of Exercise Science and Recreation, CUNY Lehman College, New York, United States of America; 6 The Human Performance Mechanic, New York, New York, United States of America; 7 Deporte y Entrenamiento Research Group, Departamento de Deportes, Facultad de Ciencias de la Actividad Física y del Deporte (INEF), Universidad Politécnica de Madrid, C./Martín Fierro 7, Spain; 8 Department of Physical Education, State University of Paraíba (UEPB), Campina Grande, Brazil; 9 Postgraduate Program in Physical Education, Federal University of Juiz de Fora, Brazil; 10 Exercise Science Program, Montcalm Community College, Sidney, United States of America; 11 Study and Research Group in Biomechanics and Psychophysiology of Exercise, Federal Institute of Education, Science and Technology of Rio Grande do Norte, Nova Cruz, Brazil; 12 Graduate Program in Cognitive Neuroscience and Behavior, Federal University of Paraiba, João Pessoa, Brazil; İzmir Democracy University: Izmir Demokrasi Universitesi, TÜRKIYE

## Abstract

This study aimed to compare the effects of a short-duration ischemic preconditioning (IPC) protocol with different cuff pressures on change of direction and jumping performance in elite male handball players. Twelve national-level male handball players (age:20.08 ± 3.12 years; height:1.81 ± 0.07 m; weight:77.88 ± 13.01 kg) participated in the study. Players visited the laboratory on five non-consecutive days. Following the familiarization session, each player completed four identical visits save for the cuff pressure used; cuff pressure was randomized into sham, 80% arterial occlusion pressure (AOP),100%AOP or 120% AOP with one used in each of the 2nd-5th visits. In the supine position, players underwent 3 cycles of 2 minutes of applied pressure and 2 minutes of reperfusion (total duration: 12 minutes). Ten minutes afterward, squat jumps (SJ) and countermovement jumps (CMJ) were performed in sequential order (5-minute rest between tests). Five minutes later, T-test and Zigzag test were performed (5-minute rest between tests). There was no significant difference across the IPC protocols for any of the parameters evaluated in the tests: SJ (F = 1.89; p = 0.151; η_p_^2^ = 0.146), CMJ (F = 1.40; p = 0.260; η_p_^2^ = 0.113), T-agility test (F = 0.01; p = 0.997; η_p_^2^ = 0.002) and Zigzag test (F = 0.240; p = 0.860; η_p_^2^ = 0.021). Our study found no effects of a 3x2-min IPC protocol using different IPC pressures on vertical jump and change of direction in elite male handball players. Therefore, it is premature to recommend the use of short-duration IPC protocols as a pre-exercise strategy for improving neuromuscular performance during ballistic and reactive athletic tasks in elite male handball players.

## Introduction

Handball is a professional and Olympic sport played by two teams of seven players (six field players and one goalkeeper) on a court of 40 × 20 m [1]. In terms of physical performance, handball is categorized as an intermittent, high-intensity game involving accelerations, decelerations, changes of direction, sprints, jumps, throws, and frequent body contact [2]. Recent rule changes (e.g., throw-off not on the line but inside the center circle) and constant improvements in the tactical use of unlimited substitutions (e.g., goalkeeper substitution rule) have increased the pace and intensity of play, leading to more attacks and goals per match [3]. Consequently, modern handball demands high levels of endurance, strength, and power to sustain repeated explosive actions throughout a game [3,4]. Additionally, key athletic tasks including jumping, throwing, and changes of direction are vital in both offensive and defensive positions. For example, increased jump height and flight time improve throwing success by helping offensive players evade defenders and better anticipate goalkeeper movements, while defensively they enhance blocking ability [1,5]. Agility is also paramount to handball success, with a recent systematic review finding that back positions (left back, center back, and right back) perform 30–40 changes of direction per game as they build their team’s positional attack [6].

Coaches can employ different training methods to increase technical, tactical, and physical performance during competitions [1]. Additionally, in recent years there has been increased interest in enhancing physical performance by targeted pre-exercise strategies beyond a typical warm-up. One such strategy, ischemic preconditioning (IPC), is a method that involves placing blood pressure cuffs or other tourniquet-type devices on the proximal arms and/or legs and performing cycles of cuff inflation and deflation, resulting in partial or full occlusion of blood flow to/from downstream muscles prior to performing an exercise bout [7,8]. One mechanism attributed to IPC’s ergogenic effects is the increased blood flow and, therefore, oxygen and nutrient delivery to downstream tissues [9]. Additionally, better tolerance of hypoxia and positive changes in perceived effort are thought to explain some of the ergogenic effects of IPC in certain exercise settings [8,10]. However, recent reviews [8,11] provide mixed evidence of ergogenic effects from IPC administration prior to exercise, noting potential differences according to participant fitness level (i.e., athletes vs. non-athletes), IPC protocol (i.e., number of occlusion and reperfusion cycles, duration of cycles, cuff pressures), and outcomes of interest (i.e., sport-specific movements vs. generalized power/speed tests). The majority of IPC research has employed protocols consisting of 3–4 sets of a 5-minute occlusion and 5-minute reperfusion at high cuff pressures, resulting in long (30 + minute) protocols and potential participant discomfort that may render such protocols infeasible in many sport settings [12,13] Given the heterogeneity in IPC response, there have been calls for the testing of alternative IPC protocols to better determine in whom, with which protocol parameters, and for what outcomes IPC has the most ergogenic benefit [11] while also accounting for practical considerations (e.g., duration) to make protocols feasible with competition preparation [14].

Several recent studies have found ergogenic benefits using shorter IPC protocols. One study in endurance runners found improved 2.4 km time-trial performance following a single 10-minute IPC protocol at a relatively low cuff pressure (~65% of arterial occlusion pressure [AOP]) [15]. A study in association football players revealed similar findings, noting improvements in endurance run times with a single 5-minute occlusion using cuff pressures of 50% and 75% of AOP [16]. Two studies examining bench press performance found an ergogenic benefit of IPC using a single 5-minute occlusion at either 100% of AOP or a consistent 170 mmHg pressure [17,18]. Finally, a study examining rock climbing performance found improved endurance following five cycles of 2-minute occlusion with 2-minute reperfusion using a cuff pressure well above AOP (>300 mmHg) [14]. These positive, time-efficient protocols are promising in improving the feasibility of using IPC in sport settings. Still, more work is needed to continue testing different IPC variables and in unique populations to determine best practices for its use.

Therefore, the purpose of this study was to investigate the effect of short-duration IPC protocol with different pressures (sham, 20 mmHg, 80-100-120% AOP) on change of directions (T-test, Zigzag test) and jump performance (squat jump, SJ and countermovement jump, CMJ) in national-level male handball players. To the best of our knowledge, this is the first study that assesses change of direction tests with jump performances after a short-duration IPC protocol with different pressures and the first IPC study testing this specific team athlete population.

## Materials and methods

### Participants

Twelve national-level male handball players (age: 20.08 ± 3.12 years; height: 1.81 ± 0.07 cm; weight: 77.88 ± 13.01 kg) participated in the study. For the eligibility criteria, subjects need to be healthy (free from any known cardiovascular or neuromuscular disorders) and able to perform all testing with full effort. G-power (G*Power 3.1.9.7, Düsseldorf, Germany) was used to determine the power of analysis. The estimated power of analysis was 0.85 when considering the obtained effect size (0.4) and a sample size of 12 participants [14,19]. Descriptive characteristics of the participants are shown in Table 1. All participants gave written informed consent, and ethical approval was obtained from the Gazi University Ethics Committee (approval number: E-77082166-604.01-994090) before the start of the study.

**Table 1:** Descriptive characteristics of the participants (n = 12).

Variables	Mean ± SD
Age (year)	20.08 ± 3.12
Height (m)	1.81 ± 0.07
Weight (kg)	77.88 ± 13.01
Muscle mass (kg)	37.58 ± 4.87
BMI (kg/m^2^)	23.93 ± 3.67
Body fat (%)	14.78 ± 6.59
Systolic blood pressure (mmHg)	124.33 ± 12.90
Diastolic blood pressure (mmHg)	70.67 ± 8.30
Right thigh circumference (cm)	56.42 ± 4.62
Left thigh circumference (cm)	56.50 ± 4.66
Arterial occlusion pressure – right thigh (mmHg)	171.25 ± 23.46
Arterial occlusion pressure – left thigh (mmHg)	168.75 ± 23.07

SD: Standard deviation

### Study design

**Fig 1:**
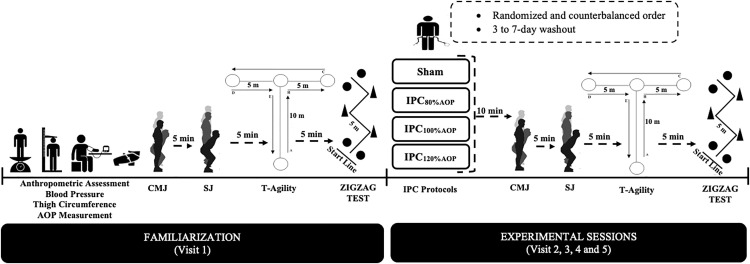
Experimental design of the study.

This study used a randomized crossover experimental design. Participants visited the laboratory on five non-consecutive days. All visits took place at a similar time period of the day to avoid circadian-induced performance changes; average lab temperatures across all visits was 21.4 ± 0.4 degrees. Visits were separated by a period of 3–7 days. The recruitment period for this study was planned between 25.07.2024 and 16.09.2024. During the familiarization (1st visit), anthropometric assessment, blood pressure, AOP measurement in supine position and thigh circumference were assessed (see Fig 1), and participants completed each of the four tests (countermovement jumps, squat jumps, T-agility, Zigzag test until they demonstrated the proper technique for familiarization purposes only. Following the familiarization (Visit 1), each participant completed four identical visits save for the cuff pressure used; cuff pressure was randomized into sham, 80% AOP, 100% AOP or 120% AOP with one used in each of the 2^nd^-5^th^ visits. Cuff pressures were blinded to the participants and participants used their regular handball shoes in all tests. For each visit, while in the supine position, participants underwent three cycles of 2 minutes occlusion followed by 2 minutes reperfusion (total duration: 12 minutes). Ten minutes afterward, CMJ and SJ were performed in sequential order (5-minute rest between tests). Five minutes later, the T-test and Zigzag test were performed (5-minute rest between tests) [8,13] (see Fig 1).

### Procedures

#### Vertical Jump Tests (Squat Jump and Counter Movement Jump).

In the SJ test, participants remained in a static position with knees bent at a 90-degree angle for approximately 2 seconds without any preliminary movement before performing a maximal effort jump. In the CMJ test, participants descended by flexing their knees approximately at 90° before reaching fully upward and jumping as high as possible. Both jumps were performed with the hands on the hips. The jumps were performed on a contact mat (Smart Jump; Fusion Sport, Brisbane, Australia) that measured the jump height based on the flight time. Each participant had five attempts per jump with 15-second intervals between attempts. The highest jump recorded for both SJ and CMJ was used for analysis. These tests were chosen for their practical relevance and high reliability [20].

#### Change of Direction and Agility Tests (Zigzag and T-Agility).

The Zigzag test was carried out on an indoor court. It consisted of four 5-m sections (total linear distance 20 m) marked with cones set at 100-degree angles (see Fig 1), requiring the participants to decelerate as quickly as possible when approaching each cone and to accelerate as quickly as possible immediately after turning around each cone. Two maximal attempts were performed with a 5-minute rest interval between attempts. Starting from a standing position with the front foot placed 0.3 m behind the first pair of timing gates (Smart Speed; Fusion Equipment, Brisbane, Australia) (i.e., starting line), the participants were instructed to complete the test as quickly as possible, until crossing the second pair of timing gates placed 20 m from the starting line [21]. The fastest time from the two trials was retained for further analysis [22].

The T-agility test was conducted on an indoor court with the timing gates (Smart Speed; Fusion Equipment, Brisbane, Australia) 0.3 m behind the starting line. Running ahead for 9.14 meters, the participants touched a cone with their hand before shifting laterally 4.57 meters to the left and touching another cone. Following that, they moved 9.14 meters to the right in lateral shuffling. Upon touching a cone and moving 4.57 meters to the left while still shuffling laterally, the participants completed the test by running 9.14 meters back toward the starting line. The test was to be completed as quickly as possible. If a participant crossed their feet or missed a cone during the sidestep phases, the test was retaken (see Fig 1). The fastest of the two trials was retained for further analysis [23].

#### Thigh circumference.

The distance from the inguinal crease to the upper part of the patella was measured using a tape measure, and a mark was made on the leg at 33% distal to the inguinal crease. To accurately represent the area where the cuffs were to be applied, the thigh circumference was measured from this mark [24,25].

#### Blood pressure.

An automatic blood pressure cuff (Omron, HEM-773) was used to measure the diastolic brachial blood pressure and systolic blood pressure. After the participants rested on their back for five minutes at room temperature, their blood pressure was measured in their left arm. The measurement was taken two times with an interval of one minute of rest between and the average was recorded in mmHg. If the systolic or diastolic blood pressure readings were not within 5 mmHg between the first two measures, a third measurement was made and the closest two values were averaged for analysis [26].

#### Determination of arterial occlusion pressure.

Thigh AOP was assessed in the supine position. An FDA-listed BFR cuff (H+Cuffs, California, US, length = 76.2 cm; width = 10.16 cm, straight version) and a hand-held 8mHz vascular Doppler (Bistos, Korea) were used to assess AOP [27,28].Participants were laid down in a supine position with blood flow restriction cuffs wrapped around the proximal part of the thighs. The Doppler was positioned on the posterior tibial artery, angled about 60˚ perpendicular to the arterial flow. The cuff was gradually inflated with increments of 20 mmHg until the sound signal emitted by the Doppler was interrupted. Subsequently, the cuff was inflated by an additional 20 mmHg and slowly deflated to confirm AOP [29]. Pressures for the IPC were then calculated as 80%, 100% and 120% AOP and were performed in a supine position three cycles of 2 min occlusion and 2 min reperfusion (total duration:12 minutes). The sham protocol was performed with the same 3 x 2-min occlusion and reperfusion cycles but with only 20 mmHg in each cuff, as has been done in past research to guard against potential placebo effects [30,31].

### Statistical analyses

Data normality and homogeneity were confirmed using the Shapiro-Wilk and Levene’s tests, respectively. One-way ANOVA with repeated measurements was used to compare test performance across IPC and sham protocols. The significance level adopted for all analyses was *p* < 0.05. Data are presented as mean and standard deviation. All analyses were performed using the Jamovi version 2.3 software. Additionally, effect sizes were calculated using Cohen’s d according to previous recommendations [32] to determine the magnitude of the differences of the Sham in relation to IPC protocols (IPC_80%-AOP_, IPC_100%-AOP_, IPC_120%-AOP_). The scale proposed by Hopkins et al. was used to categorize the effect magnitude as trivial (*d* < 0.20), small (*d* = 0.20–0.59), moderate (*d* = 0.60–1.19), large (*d* = 1.20–1.99), very large (*d* = 2.00–3.99), and nearly perfect (*d* > 4.0) [33].

## Results

Descriptive characteristics of the participants are presented in Table 1.

**Fig 2:**
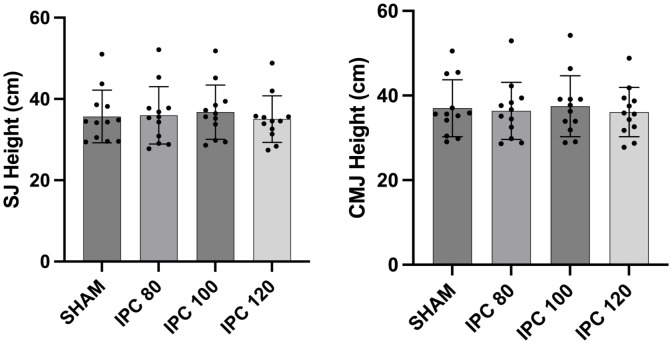
Graphical presentation of the SJ and CMJ.

**Fig 3:**
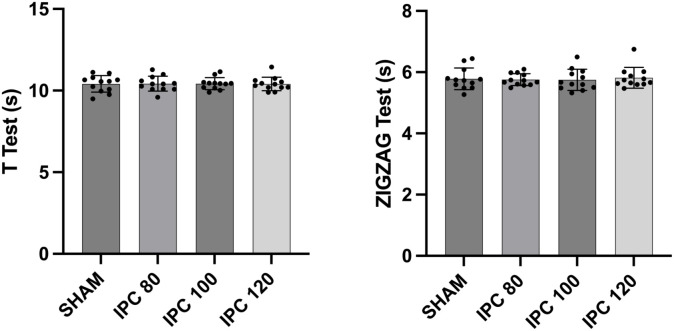
Graphical presentation of the T-Test and Zig-Zag test.

All 12 participants completed all study visits, and no adverse effects were observed during the study. Performance in each of the four tests across the four conditions is shown in Table 2. There was no significant difference across the IPC protocols for any of the parameters evaluated in the tests: SJ (F = 1.89; p = 0.151; = 0.146), CMJ (F = 1.40; p = 0.260; = 0.113), T-agility test (F = 0.01; p = 0.997; = 0.002) and Zigzag test (F = 0.240; p = 0.860; = 0.021)(see Figs 2 and 3). Effect sizes when comparing the sham protocol with each of the three IPC protocols are shown in Table 3. Effect sizes were trivial or small for all comparisons.

**Table 2:** Comparison of the performances in the vertical jumps (squat jump and countermovement jump) and change of direction tests (Zigzag test and T-test) between ischemic preconditioning (IPC) protocols.

Variables	Sham	IPC_80%-AOP_	IPC_100%-AOP_	IPC_120%-AOP_	p
SJ (cm)	35.70 ± 6.47	35.97 ± 7.07	36.75 ± 6.66	35.05 ± 5.74	0.151
CMJ (cm)	37.01 ± 6.72	36.38 ± 6.77	37.48 ± 7.20	36.09 ± 5.82	0.260
T-test (s)	10.41 ± 0.51	10.42 ± 0.46	10.43 ± 0.36	10.40 ± 0.42	0.997
Zigzag test (s)	5.79 ± 0.35	5.75 ± 0.20	5.75 ± 0.35	5.82 ± 0.34	0.860

SJ: Squat jump; CMJ: Counter movement jump; AOP: Arterial occlusion pressure.

**Table 3:** Effect size (ES) of the differences of the Sham in relation to IPC protocols.

Variables	Sham vs. IPC_80%-AOP_		Sham vs. IPC_100%-AOP_		Sham vs. IPC_120%-AOP_	
	ES	Classification	ES	Classification	ES	Classification
SJ (cm)	0.06	Trivial	0.22	Small	−0.14	Trivial
CMJ (cm)	−0.13	Trivial	0.09	Trivial	−0.20	Small
T-test (s)	0.02	Trivial	0.04	Trivial	−0.02	Trivial
Zigzag test (s)	−0.10	Trivial	−0.10	Trivial	0.10	Trivial

SJ: Squat jump; CMJ: Counter movement jump; AOP: Arterial occlusion pressure.

## Discussion

Given the need for a variety of physical attributes including speed, agility, and power to be successful in the sport of handball [2], our study tested the effectiveness of a time-efficient IPC protocol using several cuff pressures on change of direction and jump performance in elite male handball players. Overall, our results show no significant effect of any of the IPC protocols used on any of the four tests performed compared to a sham protocol. Effect sizes corroborate the ANOVA results, showing trivial or small effects of the IPC protocols compared to sham.

To our knowledge, our study is the first to examine a short duration IPC protocol as a potential ergogenic aid in the sport of elite handball. However, past studies have examined the effects of IPC on power and agility, with mixed findings. Several studies show benefits of IPC on outcomes, including 6- to 60-second sprints performed primarily on a cycle ergometer with another study showing improved 1-repetition maximum bench press exercise [34–37]. Notably, most studies were performed in healthy, active but non-athlete populations, although one study did show improvements in repeated Wingate (30-s cycle sprint) exercise in team sport athletes [38]. Positive findings have also been observed with single longer occlusions (5–10 min) for endurance running in athletes [15,16] and for bench press in recreational adults and bodybuilding athletes [17,18], as well as with a 5 x 2-min protocol in trained rock climbers [14]. In contrast, studies examining change of direction movements and/or in athlete populations tend to show little or no effect of IPC on performance outcomes. For example, a recent study by Lindner et al. found no effect of IPC applied in 3x5-min cycles on vertical jump, sprint speed, or agility in a population of collegiate athletes [39]. Similarly, Zinner et al. found no effect of 3 x 5-min IPC cycles on multidirectional sprint outcomes [40], and Gibson et al. found no effect of 3 x 5-min IPC cycles on repeated sprint cycling performance in team sports athletes [41]. Our study used a 3 x 2-min (6 total minutes of occlusion) protocol, which is a markedly smaller ischemic dose than the more common 3–4 x 5-min (15–20 min) protocols. Taken together, the literature suggests that ergogenic responses may be more likely with longer occlusion doses and in tasks emphasizing linear power/endurance, whereas shorter doses and sport-specific, change of direction demands in trained athletes often yield null effects.

Another possibility which warrants consideration is that IPC may only be effective when rest intervals between exercise bouts are relatively short. For example, studies examining the effect of IPC on repeated high-intensity exercise performances with 2–4 min rest intervals have shown improvements with IPC [42], whereas a study using 6-min rest periods [43] and our study using 5-minute rest periods between all but the repeated jumps found no such improvement in exercise performance with IPC. Among the mechanisms thought to underpin IPC’s ergogenic effects on some exercise types relate to increased vasodilation secondary to increased nitric oxide release [44] as well as enhanced prephosphorylation of phosphocreatine [45], one of the main energy contributors to sprint efforts. Given that near full recovery of muscle phosphocreatine following exhaustive exercise may take 5–6 minutes [46], enhancement of this system with IPC may be more likely to show up with shorter rest intervals but not with longer ones. Further research should seek to further elucidate the mechanisms behind IPC’s ergogenic effects to better understand which types of exercise, and in which populations, it is likely to be most effective.

Our study has several strengths, notably the sham protocol and randomized crossover design ensuring that player expectations or characteristics did not limit study findings. The study population is also unstudied and allowed us to address important questions about the efficacy of IPC in the sport of handball. However, study limitations also warrant consideration. Our sample size was small and homogenous, and our results do not necessarily apply to other populations. Second, our protocol does not use handball-specific drills/skills as performance outcomes nor was performed during/after a practice or competition, so it is possible that IPC may have different effects in such settings than in the controlled environment in which we conducted our study. Third, this study did not include a reliability analysis (i.e., test-retest) to calculate the standard error of measurement (SEM) and the smallest detectable difference (SDD) and, consequently, it remains unclear whether changes in handball players’ performance, due to a short-duration IPC protocol, are real or simply a result of testing errors or biological variation. Therefore, future studies should conduct inter-session reliability assessment to determine real performance changes (i.e., ≥ SDD) from variations within the SEM. Additionally, future research could consider assessing IPC protocols of repeated administration (e.g., across multiple days) to assess chronic effects, application of a similar protocol in upper limbs due to their important contribution on several athletic tasks performed with the upper extremities (such as throwing) in handball, and testing other cuff pressures and protocols.

## Conclusions

Our study suggests that a 3 x 2-min short-duration IPC protocol using different occlusion pressures (i.e., 80%AOP, 100%AOP and 120%AOP) does not provide acute improvements for both jumping and change of direction performance in elite male handball players. Therefore, it is premature to recommend the use of short-duration IPC protocols as pre-exercise strategy for improving neuromuscular performance during ballistic and reactive athletic tasks (i.e., those requiring the ability to produce force in a short/fast stretch shortening cycle characterized by ground contact times <0.25 seconds) in elite male handball players. However, handball coaches and strength and conditioning specialists should assess individual responses to apply IPC protocols only to those players who can benefit from their implementation. Future research should explore longer IPC durations, different timing strategies, or combining IPC protocols with other pre-competitive activities (e.g., priming or post-activation potentiation protocols based on traditional strength or plyometric exercises), as it may produce different outcomes. In addition, future studies should examine if IPC protocols improve the player’s subjective perception of readiness to compete and if chronic administration of IPC in highly trained populations yields performance or metabolic benefits.
